# Directional Forgetting for Stable Co-Adaptation in Myoelectric Control

**DOI:** 10.3390/s19092203

**Published:** 2019-05-13

**Authors:** Dennis Yeung, Dario Farina, Ivan Vujaklija

**Affiliations:** 1 Department of Electrical Engineering and Automation, Aalto University, 02150 Espoo, Finland; 2 Department of Bioengineering, Imperial College London, London SW7 2AZ, UK; d.farina@imperial.ac.uk

**Keywords:** co-adaptation, directional forgetting, electromyography, myoelectric control, upper-limb prostheses

## Abstract

Conventional myoelectric controllers provide a mapping between electromyographic signals and prosthetic functions. However, due to a number of instabilities continuously challenging this process, an initial mapping may require an extended calibration phase with long periods of user-training in order to ensure satisfactory performance. Recently, studies on co-adaptation have highlighted the benefits of concurrent user learning and machine adaptation where systems can cope with deficiencies in the initial model by learning from newly acquired data. However, the success remains highly dependent on careful weighting of these new data. In this study, we proposed a function driven directional forgetting approach to the recursive least-squares algorithm as opposed to the classic exponential forgetting scheme. By only discounting past information in the same direction of the new data, local corrections to the mapping would induce less distortion to other regions. To validate the approach, subjects performed a set of real-time myoelectric tasks over a range of forgetting factors. Results show that directional forgetting with a forgetting factor of 0.995 outperformed exponential forgetting as well as unassisted user learning. Moreover, myoelectric control remained stable after adaptation with directional forgetting over a range of forgetting factors. These results indicate that a directional approach to discounting past training data can improve performance and alleviate sensitivities to parameter selection in recursive adaptation algorithms.

## 1. Introduction

Surface electromyography (EMG) offers a non-invasive window to the peripheral nervous system (PNS) and has been used as the control input for powered prostheses since the 1950s [1,2]. In particular, myoelectric devices have been marketed towards upper-limb amputees with the appeal of providing partial functional restoration of the affected limb whilst retaining anthropomorphic aesthetics. However, most clinically available devices still employ a simplified control scheme which restricts operation to highly unintuitive sequential activation of degrees-of-freedom (DoF). Contractions from an agonist–antagonist muscle pair drives device operation along one DoF while mode-switching to other DoFs is toggled via co-contraction or pulsing [3].

More sophisticated interpretations of residual muscle activity based on pattern-recognition (PR) have since been investigated [4]. Using features (in time or frequency domain, or a combination of both) extracted from multi-channel EMG data, a repertoire of analytically distinguishable contraction patterns can be learned by the system. This allows amputees to access different prosthetic functions without switching modes while controlling actuation speeds based on contraction intensities. This control scheme has been shown to require lower cognitive load to operate and outperforms traditional direct control in online tests [5,6]. Despite these reported advantages, PR control is ultimately inconsistent with natural limb function [7]. While natural movements rely on concurrent actuation over multiple DoFs, the discrete output approximations offered by PR restricts prosthesis control to sequential operations. Simultaneous DoF activations can in principle be achieved by increasing the number of classes trained or by employing multiple classifiers in various topologies [8,9], but these methods compromise on classification accuracy and system scalability [10].

These drawbacks pertaining to the lack of simultaneous control are addressed in regression-based methods. By relying on a continuous mapping between EMG feature space and prosthesis function, simultaneous and proportional control (SPC) over multiple DoFs is enabled [7]. Such a mapping between residual muscle activations and controller output can be established by regressing the EMG characteristics of phantom limb commands to either the mirrored kinematics of the healthy limb [11,12], or to the position of a virtual cursor cueing the phantom movements [13,14,15].

In contrast to PR, where changes in muscle activation strategies can only be noticed when a decision boundary between classes is already crossed, a continuous mapping employed by regressors provides uninterrupted feedback. As such, regression-based controllers are more responsive to user adaptation as corrective measures are immediately reflected [16]. For this reason, users can partly compensate for artificially induced signal non-stationarities when using a regression-based control, contrary to a PR-based controller [17]. This characteristic is particularly advantages as environmental factors are known to perturb signal statistics. Muscle fatigue, perspiration, electrode displacement and even limb positioning contribute to the non-stationarity of recorded EMG resulting in the gradual deterioration of controller performance [18,19,20]. Although user adaptation allows partial compensation for these effects, it is thought that adaptive systems can offer a more robust and less cognitively demanding solution. Such systems learn from newly acquired data as the old model becomes defective. However, this has mostly been studied with PR-based controllers [21,22,23,24,25,26,27] and there are only a few examples of adaptive regression-based controllers [28,29,30]. In both cases, machine adaptation may occur either concurrently with user adaptation in real-time or can be implemented as incremental steps in learning.

Recently, Hahne et al. demonstrated improved myoelectric control performance with congenital amputees through a co-adaptive learning approach using recursive least squares [29]. However, adaptation stability is shown to be highly sensitive to the forgetting factor. Adaptation with certain forgetting rates results in “estimator wind-up” [31] where past data are discarded in such a way that the model overfits to new training data. In the most severe cases, this has resulted in subjects losing the ability to navigate the majority of the solution space. Various solutions to this challenge have been proposed in the form of time-varying forgetting factors [32] as well forgetting that is non-uniform in space [33]. With the latter approach, only past data in the direction of new data are discounted, meaning that only obsolete and replaceable information is forgotten.

In this study, we hypothesised that the overall performance and the instability issues encountered using the classic exponential forgetting scheme can be addressed by implementing a directional forgetting scheme. This was verified experimentally by conducting online evaluations of co-adaptation efficiency and stability with directional forgetting over a wide range of forgetting factors. Classic exponential forgetting using the generalised best performing forgetting factor reported in [29] was also tested, allowing for comparisons to be drawn.

## 2. Materials and Methods

### 2.1. Subjects

Five able-bodied subjects with no prior experience of myoelectric control participated in the study: one left-hand dominant, female, aged 20 and four right-hand dominant subjects, two female and two male aged 22–27. All subjects gave their written informed consent. This study was performed in compliance with the Declaration of Helsinki and approved by the Imperial College Research Ethics Committee in London, UK (ICREC ref:18IC4685).

### 2.2. Setup and Data Acquisition

**Figure 1:**
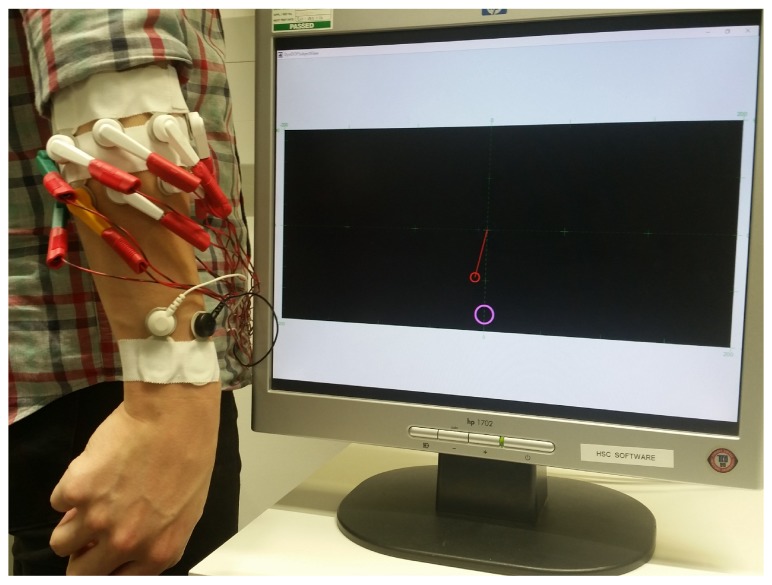
Experimental setup showing electrode position and visual feedback of the virtual task space. The red cursor is controlled via the myoelectric interface while the pink circle represents the target.

During the experiments, subjects were seated in front of a monitor with both arms relaxed by their sides. 16 monopolar sEMG channels were acquired using pre-gelled electrodes (Neuroline^®^ 720, Ambu, Denmark) placed around their dominant forearm in two rings. The electrode centres of the proximal ring were located approximately 3 cm below the lateral epicondyle of the elbow while the distal ring was adjacent just below. Horizontal distances between the centres of adjacent electrodes ranged 2.5–3.5 cm depending on the size of the subject’s forearm. Two additional gelled electrodes were attached just above the wrist of the dominant arm near the radial and ulnar styloid processes as references for the pre-amplifier and bio-amplifier. This configuration allowed for SPC over two DoFs without the need for targeted electrode placement and was aligned to that of past studies involving SPC where electrode placement is not targeted [15,16,29,34,35] and an example can be seen in Figure 1.

The detected signals were pre-amplified by five and then further amplified with a gain of either 500 or 1000 (amplifier EMGUSB2+, OT Bioelettronica, Italy), and sampled at 2048 Hz. The amplifier filtered the signals in the 10–500 Hz band. All subsequent software functionalities including signal processing, offline training and online testing were carried out using a custom MATLAB-based framework.

The acquired data were treated to adaptive common average filtering [36] and a fifth-order Butterworth band-pass filter with cut-offs at 20 Hz and 500 Hz [37]. Finally, a notch filter centred at 50 Hz was used to remove line noise. The filtered EMG data were processed in windows of 160 ms length that progressed in steps of 40 ms (120 ms of overlap) and the RMS values of each channel in the window were extracted as features.

### 2.3. Linear Regression

SPC can be achieved using a basic linear regression (LR) where the predicted output command, across a number of DoFs, is calculated as the instantaneous linear mixture of the input features:

(1) $$\hat{y}\left(t\right)=W^{⊺}x\left(t\right)$$

where $\hat{y}\left(t\right)$ is a column vector with each element corresponding to a single DoF, W is a weight matrix and x(t) is the input feature vector. Initialisation of this model involves finding the matrix W which minimises the sum-squared errors of training samples, as shown in Equation (2), where the batch nature of model initialisation has been reflected with sample number *n*. This was found analytically using the Moore–Penrose pseudo-inverse method shown in Equation (3).

(2) $$\epsilon =\sum_{n=1}^{N}{\left(y\left(n\right)-W^{⊺}x\left(n\right)\right)}^{2}$$

(3) $$W={\left({\mathrm{XX}}^{⊺}\right)}^{-1}{\mathrm{XY}}^{⊺}$$

A biasing input was also incorporated to allow for an offset of the solution plane and, as such, x(t) is prepended with a unity element and *W* is expanded with an additional row. Hence, Y is a 〈M×N〉 matrix of target labels (visual cue coordinates) and X is a matrix of training features of dimensions 〈(C+1)×N〉. *M* denotes the number of controllable DoFs, *C* is the number of EMG channels and *N* is the number of training samples. The instantaneous estimation of the command output (before post-processing) is simply obtained by solving Equation (1).

### 2.4. Recursive Least Squares with Exponential Forgetting

To facilitate model adaptation in real-time, the batch method of Equation (3) needs to be resolved. However, this is resource intensive due to the linear scaling of computational complexity with the number of training samples. Here, the recursive least-squares (RLS) algorithm may be deployed instead. Namely, as new data are obtained, the algorithm utilises past results to efficiently compute an updated least-squares estimation of the regression model parameters [31]. RLS with exponential forgetting (RLS-EF) extends the algorithm by exponentially discounting past data with each update, thus allowing for new system dynamics to override old data. This is done via the inclusion of a forgetting factor λ to the cost function in Equation (2), resulting in:

(4) $$\epsilon =\sum_{t=0}^{T}\lambda^{T-t}{\left(y\left(t\right)-W^{⊺}x\left(t\right)\right)}^{2}$$

where the notation of sample number *n* has been replaced with time *t* to reflect online implementation. Smaller values of λ correspond to a heavier discounting of past data while a value of 1 gives the “growing window” RLS algorithm where all data, new and old, are equally weighted.

The following set of update equations may then be executed to optimise the cost function (Equation (4)) as new data become available:

(5) $$\begin{array}{c} a\left(t\right)=y^{⊺}\left(t\right)-x^{⊺}\left(t\right)W\left(t\right) \end{array}$$

(6) $$\begin{array}{c} g\left(t\right)=P\left(t-1\right)x\left(t\right){\left(\lambda +x^{⊺}\left(t\right)\lambda^{-1}P\left(t-1\right)\right)}^{-1} \end{array}$$

(7) $$\begin{array}{c} P\left(t\right)=\lambda^{-1}P^{⊺}\left(t-1\right)-g\left(t\right)x^{⊺}\left(t\right)\lambda^{-1}P\left(t-1\right) \end{array}$$

(8) $$\begin{array}{c} W(t+1)=W(t)+a(t)g(t) \end{array}$$

The initial weight matrix W(0) is given by the batch method of Equation (3) while the exponentially weighted inverse of the sample covariance matrix P(0), is initialised as ${\left({\mathrm{XX}}^{⊺}\right)}^{-1}$.

With each iteration of the update rules, past data retained in the information matrix $R\left(t\right)=P{\left(t\right)}^{-1}$ are uniformly down-scaled by λ and updated with new data $\left(x\left(t\right)x{\left(t\right)}^{⊺}\right)$:

(9) $$R\left(t+1\right)=\lambda R\left(t\right)+x\left(t\right)x^{⊺}\left(t\right)$$

### 2.5. Recursive Least Squares with Directional Forgetting

As an alternative to exponential forgetting, RLS can be implemented in such a way as to employ a more content related forgetting scheme. As presented in [38], selective forgetting in the direction of the new input is achieved by the decomposition of the information matrix, R(t), into $R_{1}\left(t\right)$, which represents old data that are orthogonal to the new data, and $R_{2}\left(t\right)$, which represents old data to be discounted:

(10) $$\begin{array}{c} R\left(t\right)=R_{1}\left(t\right)+R_{2}\left(t\right) \end{array}$$

(11) $$\begin{array}{c} R_{1}\left(t\right)x\left(t\right)=0\;,\;\;x\left(t\right)\neq 0 \end{array}$$

(12) $$\begin{array}{c} R_{2}\left(t\right)x\left(t\right)=R\left(t\right)x\left(t\right) \end{array}$$

(13) $$\begin{array}{c} R\left(t+1\right)=R_{1}\left(t\right)+\lambda R_{2}\left(t\right)+x\left(t\right)x^{⊺}\left(t\right) \end{array}$$

With Equations (10)–(12), $R_{1}\left(t\right)$ and $R_{2}\left(t\right)$ are not yet fully defined, however, as the new data are only of rank 1. A fair requirement would be that $R_{2}\left(t\right)$ should also be of rank 1 with the rank of $R_{1}\left(t\right)$ as C (C + 1 is the order of R(t)). With the inclusion of these constraints, a unique solution for both matrices may be found. Effecting this decomposition to the recursive algorithm gives the new update Equations (14)–(17):

(14) $$\begin{array}{c} a\left(t\right)=y^{⊺}\left(t\right)-x^{⊺}W\left(t\right) \end{array}$$

(15) $$\begin{array}{c} \bar{P}=P\left(t-1\right)+\frac{1-\lambda }{\lambda }\frac{x\left(t\right)x^{⊺}\left(t\right)}{x^{⊺}\left(t\right)P^{-1}\left(t-1\right)x\left(t\right)} \end{array}$$

(16) $$\begin{array}{c} P\left(t\right)=\bar{P}-\frac{\bar{P}x\left(t\right)x^{⊺}\left(t\right)\bar{P}\left(t\right)}{1+x^{⊺}\left(t\right)\bar{P}\left(t\right)x\left(t\right)} \end{array}$$

(17) $$\begin{array}{c} W\left(t+1\right)=W\left(t\right)+P\left(t\right)x\left(t\right)a^{⊺}\left(t\right) \end{array}$$

### 2.6. Calibration Phase

Each experiment started with the calibration phase during which training data for the base LR model were collected. Subjects performed three repetitions of single DoF motions that corresponded to a visual cue shown on the monitor. Starting from the centre of the task space, the cue first travelled to the right of the screen, stayed for 1.5 s then returned to the origin after which it travelled to the left of the screen and dwelled for another 1.5 s before moving back to the origin. These horizontal movements were executed three times, after which three repetitions of the same nature were preformed in the vertical directions. During these cue movements, subjects were asked to match the horizontal displacement of the cue proportionally by performing wrist flexion/extension, and match vertical displacements with wrist abduction/adduction. The baseline regression model was then obtained using the batch initialisation method described in Section 2.3.

### 2.7. Online Myocontrol

Once regression models were trained, the online myocontrol portion of the experiment started, during which, subjects were able to manoeuvre a cursor in a virtual task space. Cursor position was initially estimated from Equation (1) with additional post-processing to improve controller performance.

Since no kinematic or kinetic measurements were taken as labels during the calibration phase, the mappings obtained from the initial open-looped training tended to be under-scaled. Each direction was therefore boosted:

(18) $$\begin{array}{c} \left[\begin{array}{c} {\hat{y}}_{1}^{\prime }\left(t\right)\\ {\hat{y}}_{2}^{\prime }\left(t\right) \end{array}\right]=\left[\begin{array}{c} \tau_{1a}{\hat{y}}_{1}\left(t\right)\\ \tau_{2b}{\hat{y}}_{2}\left(t\right) \end{array}\right] \end{array}$$

(19) $$\begin{array}{c} \mathrm{where}:\;\;\;\;\left\{\begin{array}{cc} a=1, & {\hat{y}}_{1}\left(t\right)\geq 0\\ a=2, & {\hat{y}}_{1}\left(t\right)<0 \end{array}\right.\;\;\;\;\&\;\;\;\;\left\{\begin{array}{cc} b=1, & {\hat{y}}_{2}\left(t\right)\geq 0\\ b=2, & {\hat{y}}_{2}\left(t\right)<0 \end{array}\right. \end{array}$$

Here, different gains were applied depending on the sign of the estimated horizontal (DoF 1) cursor displacement ($\tau_{11}$ and $\tau_{12}$ for positive and negative displacement, respectively). Likewise, different gains were applied for positive and negative vertical (DoF 2) estimates of the cursor ($\tau_{21}$ and $\tau_{22}$, respectively). All gains were tuned manually after the calibration phase to ensure effortless coverage of the task space. The criterion for setting gain values required subjects to be able to comfortably displace the cursor by 90 density-independent pixels (dp) in all single and combined DoF activations.

Finally, a seventh-order moving-average filter was applied, giving the post-processed controller output ${\hat{y}}^{\prime \prime }\left(t\right)$. The filter was implemented to reduce endpoint jitter and effectively smoothen the cursor movement.

### 2.8. Evaluation Runs

To gauge myoelectric control performance, target reaching exercises were conducted. Each run involved manoeuvring a cursor towards a sequence of 16 target circles with radii of 8 dp inside a task space that was 400 × 180 dp (target was <0.3% of the task space). The targets were evenly distributed in an inner and outer ring. Targets of the inner ring lied 40 dp from the origin while the outer ring had a radius of 75 dp with all targets evenly distributed about the origin to ensure a mixture of tasks requiring single and various degrees of simultaneous DoF control.

Subjects were given 10 s to complete each task and to successfully do so the cursor had to dwell within the target for 0.5 s. Between each task, subjects were prompted to relax and let the cursor return to the task space origin before the next task was presented. The sequence of targets presented to each subject was randomised across runs.

### 2.9. Adaptation Runs

**Figure 2:**
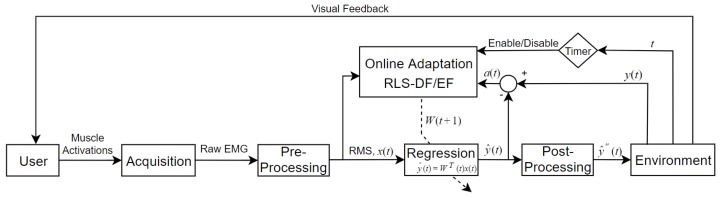
Online estimation and adaptation schematic. During adaptation runs, online adaptation with RLS was triggered if the current target was not reached within 5 s. The dotted diagonal line striking through the controller block (Regression) indicates conditional parameter update.

Concurrent adaptation of algorithm and user took place during adaptation runs where subjects attempted target reaching exercises similar to those in the evaluation runs described in the previous section. However, if the task had not been completed after 5 s, then the mapping was deemed deficient and machine adaptation was triggered. System adaptation was driven by the execution of the update rules described in Section 2.4 and Section 2.5, in which the target position and input EMG features were used to update the regression model such that the cursor converged towards the target. Machine adaptation was ceased when either the target had been reached or the task execution time had expired. A schematic of this closed-loop adaptive myoelectric controller is illustrated in Figure 2.

### 2.10. Run Sequence

**Figure 3:**
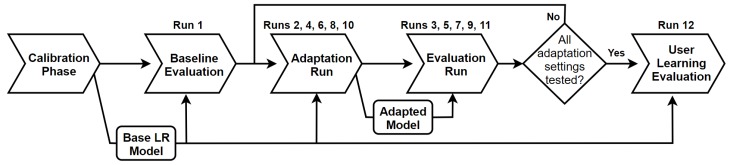
Flow diagram of the experimental procedure. The adaptation settings that were tested include: RLS-DF with λ=0.995,0.97,0.95, and 0.93, and RLS-EF with λ=0.995. The order of adaptation settings tested was randomised for each subject to prevent biasing of results.

The experiment consisted of 12 consecutive runs, which were a mixture of evaluation and adaptation runs. The sequence of assessments is shown in Figure 3. Run 1 (Baseline) was an evaluation run which gauged the baseline performance of the subject. Subsequently, the effects of machine adaptation were tested in the next 10 runs using RLS-DF using λ ranging from 0.995–0.93 and RLS-EF with λ=0.995. This was done by alternating sequences of adaptation runs (Runs 2, 4, 6, 8 and 10) and evaluations runs (Runs 3, 5, 7, 9 and 11). During an adaptation run, system adaptation was enabled with the forgetting factor and RLS variant to be tested. Each adaptation run was followed by an evaluation run. which tested the performance of the adapted model. Between each adaptation/evaluation run pair, the regression model was reverted back to the original, batch-trained condition. This was repeated until all the forgetting factors of the RLS-DF and the RLS-EF had been tested. The order of which these were tested was randomised across subjects to prevent biasing of results. The final test, Run 12 (User Learning), was an evaluation run using the base regression model which provided a reference for how much performance gain can be attributed to inherent skill improvement of the user.

Subjects were informed about the inclusion of machine adaptation prior to the adaptation runs, although no information pertaining to the actual mechanism was provided. Furthermore, pilot experiments had shown significant increases in performance by subjects between the first and second run due to the effects of learning. As such, prior to the commencement of the block of runs described earlier, a training evaluation run was conducted. This allowed participants to become familiar with myoelectric control and the virtual testing environment.

### 2.11. Performance Metrics

Four metrics were implemented to quantify each subject’s performance across the runs and such metrics have been used in past studies on myoelectric control [15,16,34]. The Completion Rate (CR) of each run is the ratio between the number of targets reached and the total number of targets. Completion Time (CT) shows the time needed to reach each individual target, Path Efficiency (PE) indicates the ratio between the optimal path from the origin to the target (straight line distance) and the actual trajectory of the cursor. Throughput (TP) is used to measure the information transfer capabilities of the human-machine interface and is calculated from a task’s index of difficulty (ID) and completion time:

(20) $$TP=\frac{ID}{CT}$$

This measure is based on Shannon’s Extension of Fitt’s Law, as presented in [39], with ID expressed as a relationship between target displacement in DoFs 1 and 2 ($D_{1},D_{2}$) and target radius (*W*):

(21) $$ID=log_{2}\left(\frac{{\left(0.5D_{1}+0.5D_{2}\right)}^{2}}{W}+1\right)$$

### 2.12. Statistical Analysis

To determine the co-adaptation stability of RLS-DF over a range of forgetting factors, the significance of performance differences between runs was calculated for all appropriate metrics. Values of TP, CT and PE for all targets were subjected to a two-way mixed ANOVA where the between-target factor was subject and the within-target factor was adaptation setting. The levels of adaptation setting include Baseline and User Learning (Runs 1 and 12, respectively) as well as RLS-DF(λ = 0.93), RLS-DF(λ = 0.95), RLS-DF(λ = 0.97), RLS-DF(λ = 0.995) and RLS-EF(λ = 0.995) (randomised amongst Runs 3, 5, 7, 9 and 11). Results from adaptation runs were not included in the analysis, as convergence to targets during those runs were machine-aided.

In the case where there was no significant interaction between the factors, the main effects were reported. If significant interaction was detected, focused Friedman Tests were conducted across subjects to detect the presence of simple effects. If the test revealed statistically significant differences between the adaptation settings, pairwise comparisons were done using the Dunn–Bonferroni test.

### 2.13. Adaptation Analysis

The resultant model from each adaptation run was compared to its base model. Changes to the model weights of the LR-based mappings were quantified using dot products between the normalised row vectors of the original model and the adapted model. A result of 1 represents no change in the contribution of the corresponding EMG channel to each DoF activation while a value of 0 represents a completely orthogonal DoF activation. The averaged dot-product value was then used to indicate the degree to which the original model was altered through online adaptation.

## 3. Results

**Figure 4:**
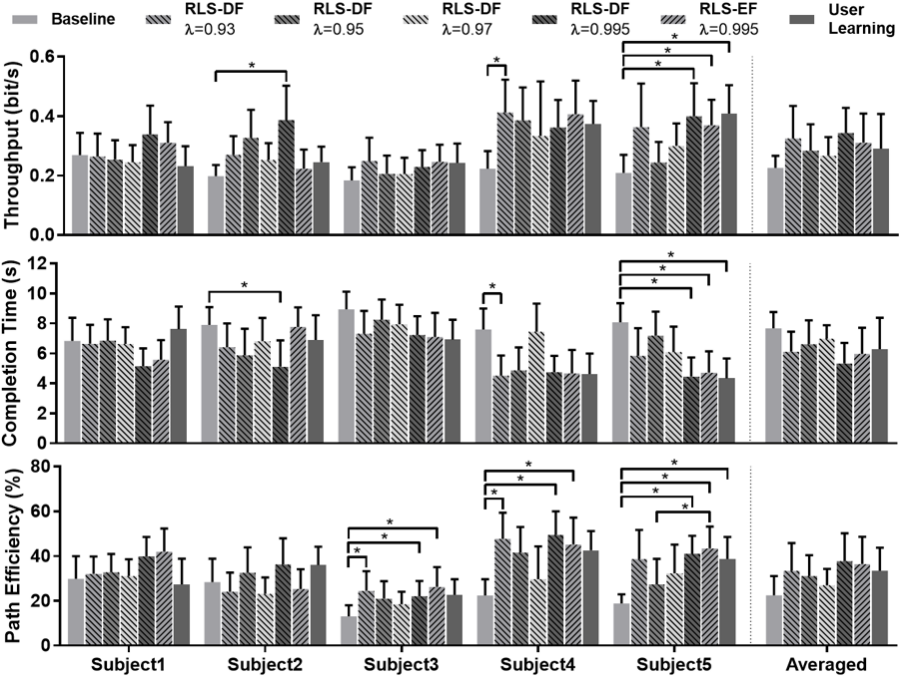
Results from the evaluation of the five subjects. * indicates statistical significance detected with Dunn–Bonferroni pairwise testing.

The overall performance results are shown in Figure 4. Two-way mixed ANOVA was conducted for TP, CT and PE. Mixed ANOVA assumes equal variances between the categories of the between-targets factor (subject) at each level of the within-targets factor (adaptation setting). This was assessed using the modified Levene’s Test for Homogeneity of variance [40] with all metrics meeting this criterion (*p* > 0.05). Variances of the differences between the levels of the repeated-measures factor was checked using Mauchly’s Test of Sphericity. Both CT (*p* = 0.371) and PE (*p* = 0.099) satisfied this assumption but TP (*p* = 0.009) failed; therefore, the Greenhouse–Geisser correction was applied to the analysis of TP.

Results from the mixed ANOVA’s indicated significant interaction between adaptation settings and subjects for all metrics (TP: F(20.925,392.340) = 2.039, *p* = 0.005; CT: F(24,450) 2.293, *p* = 0.001; PE: F(24,450) = 1.979, *p* = 0.004). As Shapiro–Wilk tests indicated non-normality of some results, the validity of interaction differences were confirmed by conducting the same testing on square-root transformations of the data which resulted in normality. As the study was mainly concerned with the adaptation stability of different forgetting factors, only the simple main effect of adaptation setting was investigated. For every subject, statistical difference between adaptation settings was checked with the Friedman Test. In the case of significance, Dunn–Bonferonni post-hoc tests were conducted with results highlighted in Figure 4. Significant differences spread across all performance metrics and all subjects except Subject 1 were detected. This occurred between “Baseline” and RLS-DF with λ=0.93 and 0.995, RLS-EF with λ=0.995 and “User Learning”. However, no significance was found in the differences between the forgetting factors of RLS-DF.

**Figure 5:**
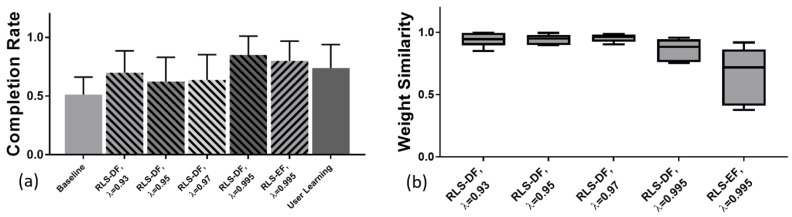
(**a**) Completion ratio of evaluation runs. Runs involving machine and user co-adaptation, regardless of algorithm or forgetting factor, had, on average, higher target hit rates compared to initial evaluations with the batch-trained model (Baseline). (**b**) Averaged dot products between normalised row vectors of weights from batch-trained models and their online-adapted versions. A value of 1 indicates no change to the sensitivities of the mapping while lower values represent larger changes to the model during machine adaptation.

No statistical testing was conducted for CR as the number of samples for each adaptation setting was limited to one per subject. As shown in Figure 5a, RLS-DF with λ=0.995 (0.85 ± 0.13) was the best performer on average followed by RLS-EF(λ=0.995) (0.80 ± 0.14). While all online-adapted models performed better compared to the initial evaluation with the batch-trained model (Baseline) (0.51 ± 0.12), performance with the same model at the end of testing “User Learning” (0.74 ± 0.16) made the online adaptation with RLS-DF, λ=0.97 (0.64 ± 0.17), λ=0.95 (0.63 ± 0.17) and λ=0.93 (0.70 ± 0.15) obsolete.

Quantification of model adaptation based on dot products is shown in Figure 5b. Here, RLS-EF was shown to induce the most changes to the model with the lowest average dot product of 0.65 and the largest standard deviation of ±0.21. Within RLS-DF, adaptation with λ=0.995 resulted in the largest changes to the weights with a mean of 0.86±0.08 while λ=0.97 showed the least change and spread with 0.96 ± 0.03.

Adaptation with RLS-DF (λ=0.995) produced models that yielded the best TP result averaged across all subjects (0.34 ± 0.06 bits/s). Similarly, RLS-DF with λ=0.995 produced the best overall results for CT (5.35 ± 0.98 s) and PE (37.75 ± 8.96%), as shown on the right-side panel of Figure 4. In comparison, the averaged TP, CT and PE results for RLS-EF with λ=0.995 were 0.31 ± 0.07 bits/s, 5.97 ± 1.26 s and 36.48 ± 8.78% respectively. Overall, RLS-DF with λ=0.995 was demonstrated to perform best in all metrics and consistently surpassed the results of RLS-EF.

## 4. Discussion

**Figure 6:**
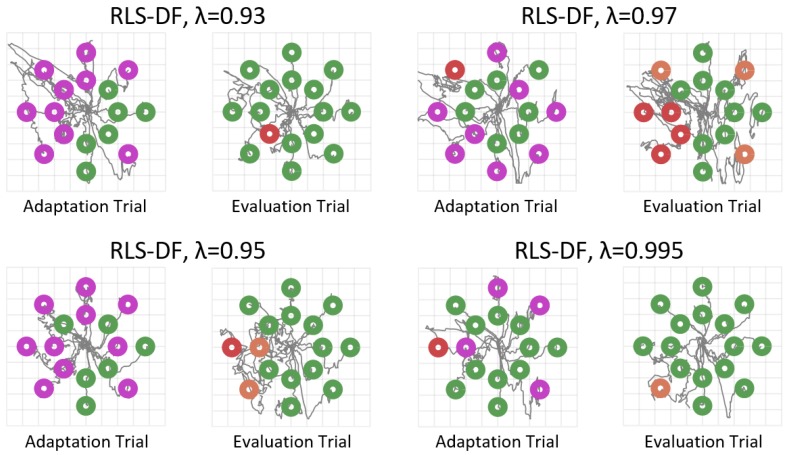
Cursor trajectories of adaptation and evaluation runs with RLS-DF from Subject 4. Green circles represent targets that were successfully reached, orange circles represent targets that were hit but dwell time was insufficient and red circles represent targets that were not hit within the time limit. Purple circles are only present in adaptation runs and represent targets that were reached with the aid of machine adaptation. Though some forgetting factors performed better than others, it can be observed that the solution space is still navigable after adaptation regardless of λ.

Directional forgetting was proposed to improve the myoelectric performance and stability of classic co-adaptive RLS algorithm. This was experimentally verified when evaluation runs of RLS-DF over a wide range of forgetting factors showed no significant decrease in performance for all subjects tested. Since the experimental set-up and timing scheme were similar to the original study by Hahne et al. on co-adaptation with RLS-EF, direct comparisons can be made between the performance of RLS-DF and the results obtained in [29] using RLS-EF. One of the most noticeable improvements then was RLS-DF’s prevention of severe over-fitting of the mapping to the target most recently adapted towards. Even with the most aggressive forgetting factor of λ=0.93, RLS-DF did not induce a complete loss in the ability to navigate the solution space (example in Figure 6), as was reported to occur with RLS-EF at λ=0.96.

When past data were uniformly forgotten, large changes were induced in the mappings, as highlighted in the model weights analyses. Here, adaptation with RLS-EF induced the most amount of change with regards to how each input element contributed to the activation of DoFs in the virtual task space. Conversely, selective discounting of past data allowed for subtler updating of model parameters, which improved stability and yielded better performance. A relatable conclusion was made in Courad et al.’s study [30], where localised and modulated updating of muscle pulling vectors in a virtual biomechanics-based model resulted in faster and more stable co-adaptative performances against perturbations when compared to co-adaptation with fixed global gains.

It is worth noting that subjects consistently performed worse in the evaluations of RLS-DF co-adaptation with λ=0.93 and 0.995. One would expect, instead, a peak in performance indicating an optimal forgetting factor with performance dropping as λ deviates from the optimal value. Conversely, experimental results show a drop in performance at moderate values of λ. A potential explanation may be that those values of λ represent adaptation which is neither aggressive nor slow enough. Given that the study set an arbitrary time limit of 5 s to deem machine adaptation as necessary for reaching a target, this may also be the time when a user would decide to discard current activation strategies in lieu of more exploratory strategies to reach the target. With an aggressive rate of machine adaptation, the user would observe a faster automatic convergence of the cursor to the target, thus, abandoning their own exploration. With more passive machine adaptation, the online mapping of exploratory activations to the target would be far less destructive while automatic convergence of the cursor to the target is still occurring. As such, this also highlights an important flaw in the current approach taken where all input is directly assigned to the target during online adaptation without regard for true user intent.

While Hahne et al. emphasised how this co-adaptation technique is well suited for enhancing the initial batch training for amputees who may experience difficulty generating combined DoF activations [29], signal non-stationarities remain a primary cause for performance degradation. Hence, future work may investigate the robustness of RLS-DF co-adaptation under non-stationary environments.

Long-term stability of myocontrol with machine adaptation was, in part, investigated by Gijberts et al. [28] who conducted offline finger force estimation in a multi-session experiment. Of particular note was the inclusion of a practical incremental learning scheme that can be initiated by the user. They used visual cues as training labels during adaptation, forgoing the need for measurement equipment. While absolute performance was inferior to closed-loop adaptation with measured ground truths, degradation was nonetheless curbed. This then raises the question of how RLS-DF adaptation for position control may perform in a similar context, where adaptation can be triggered by the user and new data labelled from visual cues are used to enable open-loop adaptation on-demand. However, in this case, machine adaptation would be incremental rather than concurrent.

The complications of mislabelling exploratory activations or providing accurate ground truths for machine adaptation can be circumnavigated by unsupervised methods [23]. Past works have shown promising results of extracting basis synergies for SPC through blind factorisation of EMG into the appropriate number ranks using non-negative factorisation (NMF) [10,34]. More recently, Lin et al. [35] demonstrated that imposing sparseness constraints to latent control primitives allows for basis information to be extracted from simultaneous DoFs activations which opens up the possibility for adaptation during arbitrary activations.

Thus far, this study indicated improved co-adaptation outcomes from implementing directional forgetting. However, further work needs to be done to truly validate the benefits of this approach with regards to actual prosthesis performance. For this claim to be made, a more rigorous study would have to be conducted involving the participation of actual end-users (amputees) performing real-world tasks. Indeed, the abstracted VR-based assessment implemented here lacks accurate representation of the physical constraints of prosthetic devices. As such, future developments would include optimisation of this approach such that it can be implemented as part of the process leading to prosthetic device use. While integration of this co-adaptive paradigm to the use of hand prostheses has already been done in [14], the study itself does not try to quantify the benefits of the co-adaptation procedure to prosthesis use.

## 5. Conclusions

This study experimentally demonstrated, on a small number of volunteers, that a more principled approach to discarding obsolete training data (RLS-DF) improves performance and co-adaptation stability over previously tested methods. Online implementation and VR based control allowed subjects to embrace the adaptive nature of the system and surpass pure user learning. However, the true advantages of the approach will be investigated in the future when a number of limb impaired participants will be recruited with an idea to test the system using a fully fitted prosthetic device. Given that the presented algorithmic extension retains a recursive nature, it remains suitable for embedded deployment and, therefore, clinical translation.
